# Phosphodiesterase 10A (PDE10A): Regulator of Dopamine Agonist-Induced Gene Expression in the Striatum

**DOI:** 10.3390/cells11142214

**Published:** 2022-07-16

**Authors:** Ryan Bonate, Gabriela Kurek, Michael Hrabak, Santanna Patterson, Fernando Padovan-Neto, Anthony R. West, Heinz Steiner

**Affiliations:** 1 Stanson Toshok Center for Brain Function and Repair, Rosalind Franklin University of Medicine and Science, North Chicago, IL 60064, USA; ryan.bonate@rosalindfranklin.edu (R.B.); gabriela.kurek@rosalindfranklin.edu (G.K.); michael.hrabak@rosalindfranklin.edu (M.H.); santanna.patterson@rosalindfranklin.edu (S.P.); 2 Discipline of Neuroscience, The Chicago Medical School, Rosalind Franklin University of Medicine and Science, North Chicago, IL 60064, USA; ferpadovan@usp.br (F.P.-N.); anthony.west@rosalindfranklin.edu (A.R.W.); 3 Center for Neurodegenerative Disease & Therapeutics, Rosalind Franklin University of Medicine and Science, North Chicago, IL 60064, USA; 4 Discipline of Cellular and Molecular Pharmacology, The Chicago Medical School, Rosalind Franklin University of Medicine and Science, North Chicago, IL 60064, USA

**Keywords:** dopamine, L-DOPA, cocaine, striatum, phosphodiesterase, cyclic nucleotide

## Abstract

Dopamine and other neurotransmitters have the potential to induce neuroplasticity in the striatum via gene regulation. Dopamine receptor-mediated gene regulation relies on second messenger cascades that involve cyclic nucleotides to relay signaling from the synapse to the nucleus. Phosphodiesterases (PDEs) catalyze cyclic nucleotides and thus potently control cyclic nucleotide signaling. We investigated the role of the most abundant striatal PDE, PDE10A, in striatal gene regulation by assessing the effects of PDE10A inhibition (by a selective PDE10A inhibitor, TP-10) on gene regulation and by comparing the basal expression of PDE10A mRNA throughout the striatum with gene induction by dopamine agonists in the intact or dopamine-depleted striatum. Our findings show that PDE10A expression is most abundant in the sensorimotor striatum, intermediate in the associative striatum and lower in the limbic striatum. The inhibition of PDE10A produced pronounced increases in gene expression that were directly related to levels of local PDE10A expression. Moreover, the gene expression induced by L-DOPA after dopamine depletion (by 6-OHDA), or by psychostimulants (cocaine, methylphenidate) in the intact striatum, was also positively correlated with the levels of local PDE10A expression. This relationship was found for gene markers of both D1 receptor- and D2 receptor-expressing striatal projection neurons. Collectively, these results indicate that PDE10A, a vital part of the dopamine receptor-associated second messenger machinery, is tightly linked to drug-induced gene regulation in the striatum. PDE10A may thus serve as a potential target for modifying drug-induced gene regulation and related neuroplasticity.

## 1. Introduction

Cyclic nucleotide phosphodiesterases (PDEs) are enzymes that degrade cyclic nucleotides (e.g., cAMP) and thus potently regulate cyclic nucleotide signaling in neurons [1]. In the rat striatum, three of the twenty-one known PDE isoforms are present at high levels, and the most abundant is PDE10A [2], which is selectively expressed by striatal projection neurons [1]. Cyclic nucleotides mediate the effects of dopamine, glutamate and other neurotransmitters in these neurons. Given the importance of striatal dopamine signaling for disorders such as schizophrenia, Parkinson’s disease, L-DOPA-induced dyskinesia and others, PDEs (including PDE10A) have been investigated as potential targets for pharmaceutical interventions in these disorders (e.g., [3,4,5]).

PDE10A is expressed in the neurons of both striatal output pathways [1]—the D1 dopamine receptor-expressing medium-sized spiny projection neurons (D1-MSNs) that make up the direct (striatonigral) pathway and the D2 receptor-expressing MSNs (D2-MSNs), which give rise to the indirect (striatopallidal) pathway. D1 receptors stimulate, and D2 receptors inhibit, second messenger signaling via their coupling to Gαolf or Gαi G-proteins, respectively, and activation or inhibition of adenylyl cyclase and cAMP formation [1,6]. PDEs by degrading cyclic nucleotides terminate such second messenger signaling. Previous studies have shown various cellular effects of PDE10A in striatal projection neurons, including the modulation of their responsiveness to glutamate inputs (e.g., [7,8]). Moreover, consistent with the importance of cyclic nucleotide signaling for gene regulation and associated neuroplasticity [9], other studies have demonstrated that experimental manipulations of PDE10A activity alter the expression of a variety of genes in these neurons—for example, genes encoding neuropeptides and transcription factors (e.g., [10,11]).

Dopamine-mediated gene regulation in the striatum (e.g., after treatment with L-DOPA, cocaine or methylphenidate) displays distinct regional variations, typically with maximal effects in the sensorimotor and lesser effects in the limbic domains [12,13,14]. In the present study, we investigated the role of PDE10A function in the gene regulation induced by these indirect dopamine agonists throughout the striatum. For one, we assessed whether the known regional variations in gene induction in the striatum were related to the magnitude of local PDE10A expression. We further determined the effect of inhibiting PDE10A with a selective PDE10A inhibitor, TP-10, at a dose that has prominent effects on cortically-evoked responses in striatal projection neurons [7,8], on the gene regulation in these neurons. Our results demonstrate a highly significant positive correlation between local PDE10A expression and the PDE10A inhibitor-induced expression of gene markers in both D1- and D2-MSNs, and a similar positive relationship between the basal PDE10A levels and gene induction by dopamine depletion and L-DOPA treatment in a Parkinson’s disease model, as well as by psychostimulants (cocaine, methylphenidate) in the intact striatum.

## 2. Materials and Methods

### 2.1. Experimental Design

Experiment 1 investigated the effects of repeated treatment with L-DOPA, either alone or in combination with the PDE10A inhibitor TP-10, on the expression of gene markers in an intact and dopamine-depleted striatum. To that end, adult rats received unilateral 6-OHDA lesions and were treated with these drugs daily for 4 weeks, starting 4 weeks after the 6-OHDA infusion. In Experiment 2, to determine the generality of our findings with the indirect dopamine agonist L-DOPA across drugs and ages, we investigated the effects of other indirect dopamine agonists, cocaine and methylphenidate (psychostimulants that act as reuptake blockers [12]), in adolescent rats. These animals received a single drug injection, and gene markers induced by acute psychostimulant treatments [12] were assessed.

### 2.2. Animals

Adult (240–260 g at the beginning of the study; Harlan, Madison, WI, USA; Experiment 1) and adolescent (75–99 g; Experiment 2) male Sprague–Dawley rats were housed with 2–3 per cage under standard laboratory conditions (12:12 h light/dark cycle; lights on at 07:00 h; food and water available ad libitum). Experiments were performed between 13:00 and 17:00 h. Prior to the treatment, the rats were allowed at least 1 week of acclimation, during which they were repeatedly handled. All procedures met the NIH guidelines for the care and use of laboratory animals and were approved by the Rosalind Franklin University Animal Care and Use Committee (protocol # 17-05; approved 19 April 2017).

### 2.3. 6-OHDA Lesions

The procedures used to produce 6-OHDA lesions (Experiment 1) have previously been described in detail [14]. In short, deeply anesthetized rats received an infusion of either 6-OHDA (6-OHDA HBr, Sigma-Aldrich; 8 μg/4 μL, in saline containing 0.1% ascorbic acid) or the vehicle (sham lesion) into the right medial forebrain bundle (coordinates, in mm: AP −4.3 (from bregma), ML 1.6, DV −8.3 (from dura) [15]). The animals included in this study had a loss in their striatal tyrosine hydroxylase immunoreactivity of 90.9 ± 1.4% (mean ± SEM) [14].

### 2.4. Drug Treatment

*Experiment 1*: A sham lesion group (S/V) received repeated injections of the vehicle (10% Cremophor in 0.9% saline, 2 mL/kg, i.p., Sigma-Aldrich, St. Louis, MO, USA; followed 30 min later by saline). The 6-OHDA-lesioned rats were treated with either the vehicle (6/V), L-DOPA (5 mg/kg, Alfa Aesar, Tewksbury, MA, USA; coadministered with 12.5 mg/kg benserazide hydrochloride, Sigma-Aldrich) (6/LD) or TP-10 (3.2 mg/kg, Pfizer Global Research and Development [7,8]), followed 30 min later by L-DOPA (6/LD + TP) (n = 8–11 each). Animals received these drug treatments once daily, 5 days per week (Mon–Fri) for 3 weeks. In week 4, rats were treated on 3 days and were killed 1 h after the final treatment. The behavior of the animals was observed for 3 h after the L-DOPA administration. Specifically, L-DOPA-induced dyskinesias were assessed (see [14] for detailed descriptions of these behaviors and their quantification). All rats with such dopamine lesions developed mid- to high levels of L-DOPA-induced dyskinesias during the repeated L-DOPA treatment (see [14]), similar to our previously reported findings after such treatments [16]. In animals co-treated with TP-10 (6/LD + TP), dyskinesias were partly inhibited (in preparation).

*Experiment 2*: The rats received a single injection of cocaine (cocaine hydrochloride, Sigma-Aldrich; 25 mg/kg, i.p.; in 0.02% ascorbic acid), methylphenidate (methylphenidate hydrochloride, Sigma-Aldrich; 5 mg/kg, i.p.) or the vehicle (n = 7–9 each) and were killed 40 min later.

### 2.5. Tissue Preparation and In Situ Hybridization Histochemistry

The rats were killed with CO_2_, and the brains were rapidly removed, frozen in isopentane cooled on dry ice and then stored at −30 °C until cryostat sectioning. Coronal sections (12 µm) were thaw-mounted onto glass slides (Superfrost/Plus, Daigger, Wheeling, IL, USA), dried on a slide warmer and stored at −30 °C. In situ hybridization histochemistry was performed as described before [17,18]. Oligonucleotide probes (48-mers; Invitrogen, Rockville, MD, USA) were labeled with [33P]-dATP. The probes had the following sequence: enkephalin, complementary to bases 436–483, GenBank accession number M28263; dynorphin, bases 862–909, M10088; substance P, bases 128–175, X56306; 5-HT1B (Htr1b), bases 62–109, NM 022225; 5-HT2C (Htr2c), bases 363–410, NM 012765; zif268 (NGFI-A, EGR-1), bases 352–399, M18416; and PDE10A, bases 541–588, NM 022236. Hybridization and washing procedures were as reported [18,19]. The sections were apposed to X-ray film (BioMax MR-2, Kodak) for 2–11 days.

### 2.6. Analysis of Autoradiograms

**Figure 1:**
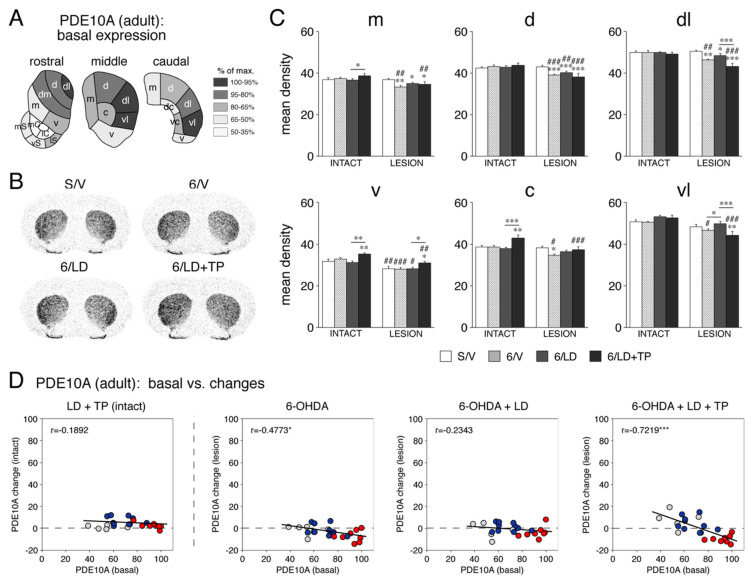
Basal expression (i.e., in S/V group, intact side) of PDE10A and changes in PDE10A expression after dopamine depletion and repeated treatment with L-DOPA and the PDE10A inhibitor TP-10 in the striatum of adult rats. (**A**) The topography of basal PDE10A expression in the adult striatum is illustrated by maps showing the distribution of PDE10A mRNA levels across the 23 sectors of the “rostral”, “middle” and “caudal” striatum in sham controls (S/V group) on the side contralateral to the infusion (intact side). The data are expressed relative to the maximal level observed (% of max.) and are coded as indicated. The sectors are largely based on the distribution of corticostriatal inputs (see [19]), which define functional domains. Limbic sectors (nucleus accumbens), rostral: medial shell (mS), ventral shell (vS), lateral shell (lS), medial core (mC), lateral core (lC); associative sectors, rostral: dorsomedial (dm), medial (m), ventral (v); middle: medial (m), central (c), ventral (v); caudal: medial (m), dorsal central (dc), ventral central (vc), ventral (v); sensorimotor sectors, rostral: dorsolateral (dl), dorsal (d); middle: dorsal (d), dorsolateral (dl), ventrolateral (vl); and caudal: dorsal (d), dorsolateral (dl), ventrolateral (vl). (**B**) Illustrations of film autoradiograms depict the expression of PDE10A mRNA in sections from the “middle” striatal level in sham controls (S/V), after a 6-OHDA lesion (right hemisphere) followed by vehicle treatment (6/V), after dopamine depletion followed by repeated L-DOPA treatment (5 mg/kg/day, 4 weeks; 6/LD) or after dopamine depletion followed by repeated treatment with L-DOPA plus the PDE10A inhibitor TP-10 (3.2 mg/kg/day, 4 weeks; 6/LD + TP). Animals were killed 60 min after the last injection. The maximal hybridization signal is in black. (**C**) Changes in the expression of PDE10A (mean density values, mean ± SEM) in rats of the S/V, 6/V, 6/LD or 6/LD + TP groups are given for the medial (m), dorsal (d), dorsolateral (dl), ventrolateral (vl), central (c) and ventral (v) sectors on the side ipsilateral (LESION) and contralateral (INTACT) to the lesion, on the middle striatal level. * *p* < 0.05, ** *p* < 0.01, *** *p* < 0.001 vs. S/V or as indicated; # *p* < 0.05, ## *p* < 0.01, ### *p* < 0.001 vs. same group on intact side. (**D**) The relationship between basal expression of PDE10A in adults and changes in PDE10A expression after these treatments is shown. The scatter plots depict basal PDE10A expression (S/V, intact side; in % of max.) versus changes in PDE10A expression after L-DOPA + TP-10 treatment (LD + TP group) on the intact side (left), changes after dopamine depletion only (6-OHDA; 6/V group, lesion side) (second from left), changes after dopamine depletion plus L-DOPA treatment (6-OHDA + LD; 6/LD group, lesion side) (second from right) and changes after dopamine depletion plus L-DOPA + TP-10 treatment (6-OHDA + LD + TP, 6/LD + TP group, lesion side) (right), all expressed as the percentage of maximal values in the basal expression (S/V, intact side) for the eight sensorimotor sectors (red dots), the 10 associative sectors (blue dots) and the 5 limbic sectors (gray dots). * *p* < 0.05, *** *p* < 0.001.

Gene expression in the striatum was assessed in sections from 3 rostrocaudal levels (rostral, approximately at +1.6 mm relative to bregma [15]; middle, +0.4; caudal, −0.8) in a total of 23 sectors (Figure 1A). These sectors are mostly defined by their predominant cortical inputs and thus reflect different functional domains (see [19,20]). Eighteen of these sectors represent the caudate putamen (10 associative and 8 sensorimotor sectors), and 5 represent the nucleus accumbens (limbic sectors).

Hybridization signals on film autoradiograms were measured by densitometry (NIH Image; Wayne Rasband, NIMH, Bethesda, MD, USA), as described in [18]. The mean densities were corrected for the background by subtracting the mean density values measured over white matter (corpus callosum). The illustrations of film autoradiograms displayed are computer-generated images, and are contrast-enhanced where necessary.

### 2.7. Statistics

Treatment effects were determined by one- or two-factor ANOVA (Statistica, StatSoft, Tulsa, OK, USA). Newman–Keuls post hoc tests were used to describe the differences between individual groups. Gene regulation effects for different genes were compared using Pearson correlations. Differences were considered significant if *p* < 0.05.

The data obtained for the drug effects on the expression of dynorphin, enkephalin and 5-HT1B in the S/V, 6/V and 6/LD groups were presented in more detail in our companion paper [14]; some data are included here again for contrast with the effects in the 6/LD + TP group (treated in parallel), as well as the effects on zif268 expression.

## 3. Results

### 3.1. Experiment 1: Relationship between PDE10A Expression and the Effects of Dopamine Depletion and Repeated L-DOPA and TP-10 Treatment in Adults

#### 3.1.1. PDE10A Expression in the Striatum of Adults

The basal expression of PDE10A (i.e., in sham-lesioned animals (S/V) on the non-infused (intact) side) was mapped throughout the 23 sectors of the adult striatum (Figure 1A). The expression was highest in the lateral (sensorimotor; Figure 1D, red dots) striatum, with peaks located in the dorsolateral, lateral and ventrolateral sectors on the “rostral”, “middle” and “caudal” striatal levels, respectively. The expression was lower in the medial, central and ventral (associative; Figure 1D, blue dots) sectors and lowest in the nucleus accumbens (limbic sectors; Figure 1D, gray dots). In the latter, the PDE10A expression was highest in the lateral shell and lowest in the two core regions (Figure 1A).

Dopamine depletion by 6-OHDA and the subsequent drug treatments had modest but differential effects on the PDE10A expression across the different striatal sectors (Figure 1). In 6-OHDA-lesioned rats (6/V), the PDE10A expression on the dopamine-depleted side was statistically significantly decreased (*p* < 0.05, 6/V vs. S/V) in nine of the twenty-three sectors. This effect was most robust in the dorsal and dorsolateral sensorimotor sectors (Figure 1C,D) and in the medial shell of the nucleus accumbens. In animals repeatedly treated with L-DOPA alone following the 6-OHDA lesion (6/LD), the decrease in the PDE10A expression tended to be less pronounced (*p* < 0.05, 6/LD vs. S/V, 5/23 sectors) (Figure 1C,D). In these animals, repeated treatment with L-DOPA alone did not affect PDE10A expression on the intact side (*p* > 0.05, 6/LD vs. S/V).

In contrast, animals that received repeated TP-10 + L-DOPA treatment (6/LD + TP) showed differential changes in the PDE10A expression on the dopamine-depleted side. That is, in the rostral (ventral) striatum, the PDE10A expression was statistically significantly increased (*p* < 0.05, 6/LD + TP vs. S/V) in four of the ten rostral sectors (ventral, nucleus accumbens medial core, lateral core and lateral shell) (Figure 1D), as it was in the ventral sector on the middle level (Figure 1C,D). In contrast, the expression was significantly decreased (*p* < 0.05, 6/LD + TP vs. S/V) in 10 sectors, mostly sensorimotor, on all three rostrocaudal levels (Figure 1). A similar effect was found when the 6/LD + TP group was compared with the 6/LD group (increased expression in five rostral ventral sectors and in the middle ventral sector; decreased expression in six sensorimotor sectors; *p* < 0.05, 6/LD + TP vs. 6/LD) (Figure 1).

In contrast to the other treatments, repeated TP-10 + L-DOPA treatment (6/LD + TP) also affected the PDE10A expression on the intact side. These animals displayed increased PDE10A expression (*p* < 0.05, 6/LD + TP vs. S/V) in eight of the twenty-three sectors, mostly in associative sectors (seven of the ten associative sectors) (Figure 1C). The same was found when the 6/LD + TP group was compared with the 6/LD group (increased expression in ten sectors; nine of the ten associative sectors; *p* < 0.05, 6/LD + TP vs. 6/LD), indicating that the TP-10 treatment produced this effect.

#### 3.1.2. Dynorphin and Enkephalin Expression

**Figure 2:**
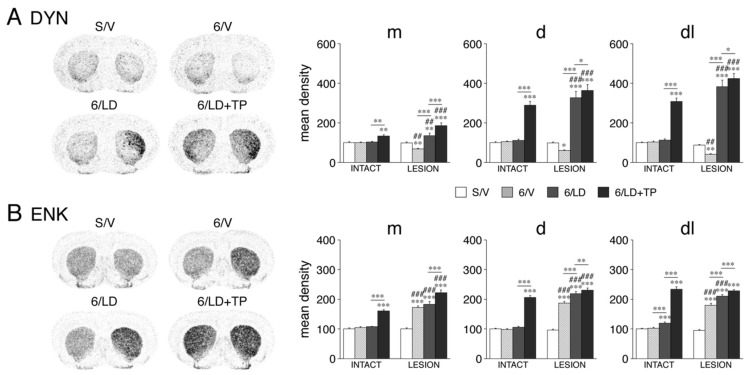
Changes in expression of dynorphin (**A**) and enkephalin (**B**) in the striatum after dopamine depletion and repeated treatment with L-DOPA and the PDE10A inhibitor TP-10 in adults. Illustrations of film autoradiograms (left) show the expression of dynorphin (DYN) and enkephalin (ENK) in sections from the mid-striatal level in sham controls (S/V), after a 6-OHDA lesion (right hemisphere) only (6/V), after dopamine depletion followed by repeated L-DOPA treatment (6/LD) or after dopamine depletion followed by repeated L-DOPA plus TP-10 treatment (6/LD + TP). Changes in expression (mean density values, mean ± SEM) (right) for dynorphin or enkephalin in rats of the S/V, 6/V, 6/LD and 6/LD + TP groups are given for the medial (m), dorsal (d) and dorsolateral (dl) sectors on the side ipsilateral (LESION) and contralateral (INTACT) to the lesion, on the middle striatal level. The values are normalized relative to values in sham controls (S/V, intact side). * *p* < 0.05, ** *p* < 0.01, *** *p* < 0.001 vs. S/V or as indicated; ## *p* < 0.01, ### *p* < 0.001 vs. same group on intact side.

We assessed the impact of these treatments on the expression of dynorphin and enkephalin as markers for D1-MSNs and D2-MSNs, respectively [21]. The effects of the dopamine depletion and subsequent repeated L-DOPA treatment (groups 6/V and 6/LD) on these neuropeptides have been reported in detail before [14] and are summarized here. Consistent with a large amount of literature (see [14]), the 6-OHDA lesion (6/V) produced a decrease in the dynorphin expression (*p* < 0.05, 6/V vs. S/V, 16/23 sectors) and an increase in the enkephalin expression (*p* < 0.05, 21/23 sectors) (Figure 2). Also consistent with the literature, repeated L-DOPA treatment after the 6-OHDA lesion (6/LD) produced an increase in the dynorphin expression (*p* < 0.01, 6/LD vs. S/V, 16/23 sectors; 6/LD vs. 6/V, 23/23 sectors) and a further increase in the enkephalin expression (*p* < 0.01, 6/LD vs. S/V, 22/23 sectors; 6/LD vs. 6/V; 15/23 sectors) (Figure 2). On the intact side, after repeated treatment with L-DOPA alone (6/LD), there were minimal increases in the dynorphin (*p* < 0.05, 6/LD vs. S/V, 2/23 sectors) and enkephalin (5/23 sectors) expression, all in sensorimotor sectors (Figure 2).

Animals that received repeated TP-10 + L-DOPA treatment (6/LD + TP) displayed greatly increased expression of dynorphin and enkephalin in the striatum of both hemispheres (Figure 2). For dynorphin (Figure 2A), the expression was increased on the dopamine-depleted side (*p* < 0.05, 6/LD + TP vs. S/V) in 23 of the 23 sectors (*p* < 0.001, 20/23 sectors), an effect that was significantly greater than after L-DOPA treatment alone (*p* < 0.05, 6/LD + TP vs. 6/LD, 20/23 sectors). Similarly, on the intact side, the dynorphin expression was increased (*p* < 0.01, 6/LD + TP vs. S/V) in 17 of the 23 sectors, an effect that was also significantly greater than after L-DOPA treatment alone (*p* < 0.01, 6/LD + TP vs. 6/LD, 16/23 sectors). However, the increase in dynorphin expression on the lesion side was greater than that on the intact side (*p* < 0.01, 6/LD + TP, lesion vs. intact, 21/23 sectors).

The situation was similar for enkephalin (Figure 2B). On the dopamine-depleted side, the enkephalin expression was increased (*p* < 0.001, 6/LD + TP vs. S/V) in 23 of the 23 sectors, an effect that was significantly greater than after L-DOPA treatment alone (*p* < 0.01, 6/LD + TP vs. 6/LD, 15/23 sectors). On the intact side, the enkephalin expression was increased (*p* < 0.001, 6/LD + TP vs. S/V) in 20 of the 23 sectors, and this effect was also significantly greater than after L-DOPA treatment alone (*p* < 0.001, 6/LD + TP vs. 6/LD, 20/23 sectors). Again, the increase in the enkephalin expression on the lesion side was greater than that on the intact side in most sectors (*p* < 0.01, 6/LD + TP, lesion vs. intact, 17/23 sectors).

#### 3.1.3. Zif268 Expression

**Figure 3:**
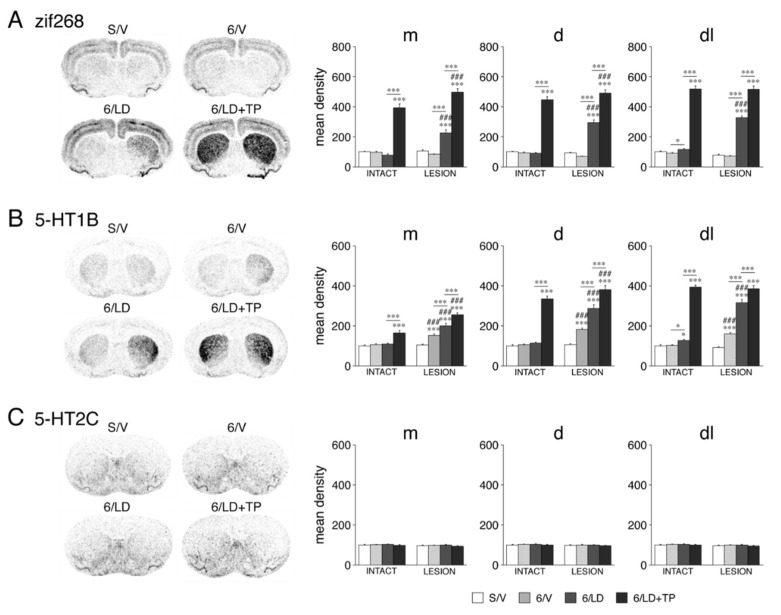
Changes in expression of the immediate-early gene zif268 (**A**) and the serotonin receptor subtypes 5-HT1B (**B**) and 5-HT2C (**C**) in the striatum after dopamine depletion and repeated treatment with L-DOPA and the PDE10A inhibitor TP-10 in adults. Illustrations of film autoradiograms (left) show the expression of zif268, 5-HT1B and 5-HT2C in sections from the middle striatum in sham controls (S/V), after a 6-OHDA lesion (right hemisphere) (6/V), after dopamine depletion followed by repeated L-DOPA treatment (6/LD) or after dopamine depletion followed by repeated L-DOPA plus TP-10 treatment (6/LD + TP). Changes in expression (mean density values, mean ± SEM) (right) for these genes in rats of the S/V, 6/V, 6/LD or 6/LD + TP groups are given for the medial (m), dorsal (d) and dorsolateral (dl) sectors on the sides ipsilateral (LESION) and contralateral (INTACT) to the lesion, on the middle striatal level. The values are normalized relative to values in sham controls (S/V, intact side). Note that zif268 and 5-HT1B, both examples of synaptic activity-regulated genes, showed pronounced changes in expression in response to these experimental treatments, while 5-HT2C was unaffected. * *p* < 0.05, *** *p* < 0.001 vs. S/V or as indicated; ### *p* < 0.001 vs. same group on intact side.

We also assessed the effects of these treatments on the expression of an immediate-early gene, zif268, which encodes a transcription factor and is induced by various drugs and other treatments. The 6-OHDA lesion alone (6/V) did not produce statistically significant changes in the zif268 expression (*p* > 0.05, 6/V vs. S/V, 23/23 sectors) (Figure 3A). Repeated L-DOPA treatment after the 6-OHDA lesion (6/LD) increased the zif268 expression (*p* < 0.01, 6/LD vs. S/V) in 19 of the 23 sectors. In contrast, on the intact side, repeated treatment with L-DOPA alone (6/LD) did not affect the zif268 expression (*p* > 0.05, 6/LD vs. S/V, 23/23 sectors).

In contrast to L-DOPA alone, repeated treatment with TP-10 + L-DOPA (6/LD + TP) produced massive increases in the expression of zif268 on both sides (Figure 3A). On the dopamine-depleted side, the expression was increased (*p* < 0.001, 6/LD + TP vs. S/V) in 23 of the 23 sectors. This effect was significantly greater than that after L-DOPA treatment alone (*p* < 0.001, 6/LD + TP vs. 6/LD) in all 23 sectors. On the intact side, the zif268 expression was also increased (*p* < 0.01, 6/LD + TP vs. S/V) in all 23 sectors. However, the increase on the lesion side was again greater than that on the intact side (*p* < 0.05, 6/LD + TP, lesion vs. intact) in 17 of the 23 sectors, but not in the six lateral (sensorimotor) sectors (*p* > 0.05), on all three rostrocaudal levels (Figure 3A), which may reflect a “ceiling” effect.

#### 3.1.4. 5-HT1B and 5-HT2C Serotonin Receptors: Selective Impact on 5-HT1B Expression

We further measured the impact of these treatments on the expression of the 5-HT1B and 5-HT2C serotonin receptor subtypes [14]. The 6-OHDA lesion alone (6/V) produced significant increases in the 5-HT1B expression (*p* < 0.05, 6/V vs. S/V) in 20 of the 23 sectors (Figure 3B). Repeated L-DOPA treatment after the 6-OHDA lesion (6/LD) increased the 5-HT1B expression (*p* < 0.05, 6/LD vs. S/V) in 22 of the 23 sectors (*p* < 0.001, 19/23 sectors), and this effect was greater than after the lesion alone (*p* < 0.05, 6/LD vs. 6/V) in 16 sectors. In contrast, repeated treatment with L-DOPA alone (6/LD) produced minimal increases in 5-HT1B expression on the intact side (*p* < 0.05, 6/LD vs. S/V, 3/23 sectors).

After repeated TP-10 + L-DOPA treatment (6/LD + TP), the rats showed a greatly enhanced expression of 5-HT1B in the striatum on both sides (Figure 3B). On the dopamine-depleted side, the expression was increased (*p* < 0.05, 6/LD + TP vs. S/V) in all 23 sectors (*p* < 0.001, 21/23 sectors), and this effect was significantly greater than after L-DOPA treatment alone (*p* < 0.01, 6/LD + TP vs. 6/LD) in 19 of the 23 sectors. On the intact side, the 5-HT1B expression was increased (*p* < 0.01, 6/LD + TP vs. S/V) in 18 of the 23 sectors. However, the increase in the 5-HT1B expression on the lesion side was also greater than that on the intact side (*p* < 0.05, 6/LD + TP, lesion vs. intact) in 17/23 sectors (but not in four sensorimotor sectors, *p* > 0.05) (Figure 3B).

In stark contrast to 5-HT1B, the expression of 5-HT2C was not affected by dopamine-depletion or any of the drug treatments in any of the 23 sectors (Figure 3C).

#### 3.1.5. Relationship between Basal PDE10A Expression and the Effects of Dopamine Depletion and Drug Treatments on Gene Expression in Adults

**Figure 4:**
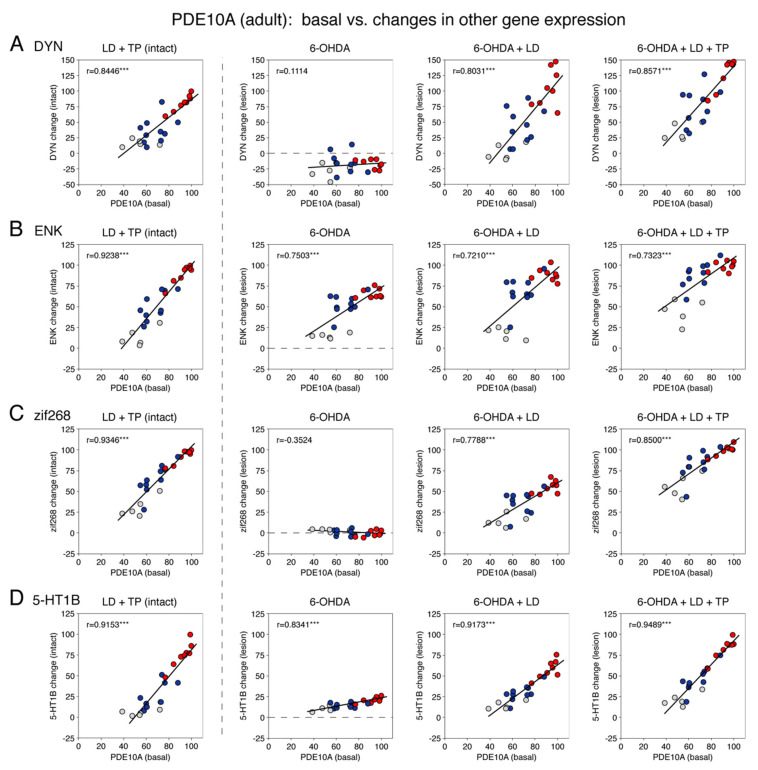
Relationship between basal expression of PDE10A and changes in the expression of dynorphin (**A**), enkephalin (**B**), zif268 (**C**) and 5-HT1B (**D**) after dopamine depletion and repeated treatment with L-DOPA and the PDE10A inhibitor TP-10 in adults. Scatter plots depict correlations between basal PDE10A expression (S/V, intact side; in % of max.) and changes in the expression of these genes after L-DOPA + TP-10 treatment (LD + TP group) on the intact side (left), changes after dopamine depletion only (6-OHDA; 6/V group, lesion side) (second from left), changes after dopamine depletion plus L-DOPA treatment (6-OHDA + LD; 6/LD group, lesion side) (second from right) and changes after dopamine depletion plus L-DOPA + TP-10 treatment (6-OHDA + LD + TP, 6/LD + TP group, lesion side) (right), all expressed as the percentage of maximal values on the intact side after L-DOPA + TP-10 treatment (LD + TP group; left) for the eight sensorimotor sectors (red dots), the ten associative sectors (blue dots) and the five limbic sectors (gray dots). *** *p* < 0.001.

We next assessed whether there was a relationship between the observed changes in gene expression and the magnitude of the basal expression of PDE10A in these 23 striatal sectors in adults (Figure 4). The basal expression was determined in the striatum of the non-infused (intact) side in our control group (S/V) (Figure 1). This analysis also highlighted the variations of effects between limbic, associative and sensorimotor sectors (Figure 4). Our findings are described as follows.

As mentioned above, the basal expression of PDE10A was highest in the sensorimotor sectors (Figure 4, red dots), intermediate in the associative sectors (blue dots) and lowest in the limbic/nucleus accumbens sectors (gray dots). As also discussed previously, all lesion- or drug-induced increases in gene expression were highest in the sensorimotor sectors, intermediate in the associative sectors and lowest in the limbic sectors. Confirming this association between the basal PDE10A expression and gene regulation, our analysis showed that all the increases in gene expression were highly positively correlated with the levels of basal expression of PDE10A in these 23 striatal sectors (*r =* 0.7210–0.9489, *p* < 0.001) (Figure 4A–D).

**Figure 5:**
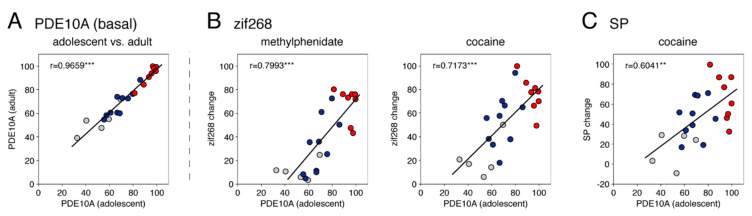
Comparison of basal PDE10A expression in adolescent and adult rats (**A**) and the relationship between basal PDE10A expression and changes in zif268 (**B**) and substance P expression (**C**) induced by acute treatment with methylphenidate or cocaine in adolescents. Scatter plots depict correlations between basal PDE10A expression in adolescents (vehicle control, in % of max.; Experiment 2) and that in adults (S/V, intact side, in % of max.; Experiment 1) (**A**), and increases in expression of zif268 and substance P (SP) after acute injection of methylphenidate (5 mg/kg; in % of max. cocaine) or cocaine (25 mg/kg; in % of max.) in adolescents (**B**,**C**), for the eight sensorimotor sectors (red dots), the ten associative sectors (blue dots) and the five limbic sectors (gray dots). ** *p* < 0.01, *** *p* < 0.001.

For example, as described above, the 6-OHDA lesion alone (6/V, lesion side) produced an increased expression of enkephalin (Figure 4B) and 5-HT1B (Figure 4D), and these increases were positively correlated with the basal PDE10A expression (*r =* 0.7503, *p* < 0.001 and *r =* 0.8341, *p* < 0.001, respectively). In contrast, the dynorphin expression (Figure 4A) was decreased after the dopamine lesion, but this decrease was unrelated to the basal PDE10A expression (*r =* 0.1114, *p* > 0.05). The zif268 expression (Figure 4C) was unaltered by the lesion alone and was thus also unrelated to the PDE10A expression (*r =* −0.3524, *p* > 0.05). 

Repeated L-DOPA treatment after the 6-OHDA lesion (6/LD) produced an increased expression of dynorphin, enkephalin, zif268 and 5-HT1B on the side of the lesion, and these increases were all positively correlated with basal PDE10A expression (*r =* 0.8031, 0.7210, 0.7788 and 0.9173, respectively; all *p* < 0.001) (Figure 4A–D).

After repeated TP-10 + L-DOPA treatment (6/LD + TP), on the dopamine-depleted side, the increased expression of dynorphin, enkephalin, zif268 and 5-HT1B were also positively correlated with the basal PDE10A expression (*r =* 0.8571, 0.7323, 0.8500 and 0.9489, respectively; all *p* < 0.001) (Figure 4A–D). Similarly, on the intact side in these animals (LD + TP), the increased expression of dynorphin, enkephalin, zif268 and 5-HT1B were all positively correlated with the basal PDE10A expression (*r =* 0.8446, 0.9238, 0.9346 and 0.9153, respectively; all *p* < 0.001) (Figure 4A-D). Indeed, this treatment (LD + TP, intact side) produced the highest correlations for these four genes (Figure 4). On the other hand, across these four treatments, increases in the 5-HT1B expression were most highly correlated with the basal PDE10A expression (*r =* 0.9153, 0.8341, 0.9173 and 0.9489, respectively; all *p* < 0.001) (Figure 4D).

### 3.2. Experiment 2: Relationship between Basal Expression of PDE10A and the Effects of Acute Cocaine and Methylphenidate in Adolescents

#### 3.2.1. PDE10A Expression in the Striatum of Adolescents

The basal expression of PDE10A in adolescents displayed a regional distribution in the striatum that was identical to that in adults (Experiment 1). Thus, across the 23 striatal sectors, the basal PDE10A mRNA levels were highly significantly correlated between adolescents (vehicle group) and adults (S/V, intact side) (*r =* 0.9659, *p* < 0.001) (Figure 5A). Similar to adults (Figure 1), the highest expression in adolescents was present in the sensorimotor sectors (Figure 5A, red dots), with peaks shifting from the dorsolateral sector on the rostral level to the ventrolateral sector on the caudal level (not shown). Again, the levels of expression were lowest in the nucleus accumbens (Figure 5A, gray dots). Acute treatment with these psychostimulants had no significant effect on the PDE10A expression (results not shown).

#### 3.2.2. Zif268 Expression and Substance P Expression

We assessed the impact of acute psychostimulant treatment on the expression of the immediate-early gene zif268 and the neuropeptide substance P, a marker for acute changes in D1-MSNs [21], in adolescents. Overall, the zif268 expression was significantly increased by cocaine (25 mg/kg) in 20 of the 23 sectors and by methylphenidate (5 mg/kg) in 15 sectors. On the other hand, substance P expression was significantly increased by cocaine in 16 sectors and by methylphenidate in 14 sectors.

The regional distribution across the striatum of acute gene induction by cocaine and methylphenidate was consistent with our previously published distribution (maps) (see [12,13] for reviews), showing maximal induction in sensorimotor sectors (Figure 5, red dots) and minor induction in the limbic sectors (Figure 5, gray dots) for both psychostimulants. Our correlation analysis showed that there was a significant positive correlation between the magnitude of zif268 induction by cocaine (*r =* 0.7173, *p* < 0.001) or by methylphenidate (*r =* 0.7993, *p* < 0.001) and the levels of basal PDE10A expression in the 23 striatal sectors in adolescents (Figure 5B). Zif268 induction by cocaine was thus also correlated with zif268 induction by methylphenidate (*r =* 0.8698, *p* < 0.001). For substance P, the cocaine-induced increases in mRNA levels were also significantly correlated with the levels of basal PDE10A expression across the 23 striatal sectors (*r =* 0.6041, *p* < 0.01) (Figure 5C). However, the methylphenidate-induced substance P mRNA levels only showed a strong tendency for a positive correlation (*r =* 0.4092, *p* < 0.06; not shown) due to relatively less robust effects on substance P expression by this low-intermediate methylphenidate dose [22].

## 4. Discussion

The present study investigated the relationship between PDE10A function and gene regulation by dopamine depletion and dopamine receptor stimulation in striatal projection neurons of adolescent and adult male rats. Our main findings are summarized as follows. (1) The inhibition of PDE10A by the selective PDE10A inhibitor TP-10 (in combination with L-DOPA) produced dramatic increases in gene expression (dynorphin (D1-MSNs), enkephalin (D2-MSNs), zif268 and 5-HT1B, but not 5-HT2C) in the striatum of adults. These increases were highly positively correlated with the regional levels of PDE10A mRNA in the normal striatum. (2) After dopamine depletion by 6-OHDA, the increases in striatal gene expression (enkephalin, 5-HT1B), but not the decreases (dynorphin), were also positively correlated with local PDE10A expression in adults. (3) After dopamine depletion plus repeated L-DOPA treatment, increases in gene expression (dynorphin, enkephalin, zif268 and 5-HT1B) were again positively correlated with basal PDE10A expression. (4) Psychostimulant (cocaine, methylphenidate)-induced increases in gene expression (zif268, substance P (D1-MSNs)) were similarly positively correlated with the basal expression of PDE10A in adolescents. (5) The basal levels of PDE10A mRNA and the increases in the expression of these gene markers were maximal in the sensorimotor and minimal in the limbic striatal sectors for both adults and adolescents. (6) Only modest changes in the expression of PDE10A itself were seen after dopamine depletion and the L-DOPA and TP-10 treatments, and these changes were in the opposite direction in the sensorimotor and limbic sectors.

### 4.1. PDE10A Expression in the Striatum: Basal Expression and Effects of Dopamine Depletion and Drug Treatments

PDE10A is the most abundant PDE isoform in the striatum [2]. Few previous studies have reported on the regional differences in the basal PDE10A expression of the rat striatum, without mapping the distribution. For example, PDE10A mRNA expression has been described as “relatively uniform” throughout the striatum [2], although somewhat higher levels of mRNA and protein are discernable in the lateral striatum in other reports (e.g., [23]). Our study is the first to provide detailed maps of the basal PDE10A mRNA expression across the rostral to caudal striatal levels. These maps clearly show a medial/ventral-to-lateral gradient with the highest mRNA levels in the lateral (sensorimotor) striatal sectors, lower levels in the medial and ventral (associative) sectors and the lowest levels in the nucleus accumbens (limbic sectors). In the latter, the expression was notably highest in the lateral part of the shell and lowest in the core. Moreover, these differential expression levels were highly correlated between adolescent and adult rats.

After the various treatments (i.e., dopamine depletion, L-DOPA and TP-10 treatment), the changes in PDE10A expression were relatively modest in comparison to the magnitudes of both the regional differences in basal expression and drug-induced changes for other genes. PDE10A is expressed in both the D1- and D2-MSNs [1], and treatment-induced differential changes between these neuron subtypes may have obscured the magnitude of effects. However, an extensive study using TRAP analysis to assess the impact of dopamine depletion and L-DOPA treatment on the expression of thousands of genes in individual neuron subtypes did not identify PDE10A as affected in either subtype (in contrast to other striatal PDEs, such as PDE1c and others) [24]. However, this analysis used whole striatal tissue extracts and was thus not designed to address potential regionally differential effects.

Indeed, another recent study was the first to report differential PDE10A regulation between the limbic striatum (nucleus accumbens) and the caudate putamen (CPU; corresponding to our associative/sensorimotor sectors) after dopamine depletion [25]. This study demonstrated decreased PDE10A mRNA and protein levels and increased cAMP levels in the CPU, and opposite changes in the nucleus accumbens (i.e., increased PDE10A protein and activity, and unchanged mRNA levels) [25] that were consistent with our findings. Our results extend these earlier findings in showing that the decreases in PDE10A mRNA levels after dopamine depletion were most robust in the sensorimotor sectors, and that a subsequent L-DOPA treatment tended to ameliorate, while the TP-10 treatment enhanced, these differential effects (i.e., the decrease in the expression in sensorimotor sectors, and the increase in limbic and some associative sectors). Future research will have to address the mechanisms underlying this differential regulation of PDE10A between limbic and non-limbic striatal regions.

### 4.2. Changes in Gene Expression in Striatum after PDE10A Inhibition

Gene expression in the striatum is driven by excitatory input (from the cortex and thalamus) and is modulated (facilitated or attenuated) by dopamine and other neurotransmitters [13]. For example, dopamine facilitates glutamate input via D1 receptor stimulation and inhibits glutamate input via D2 receptor stimulation [26]. D1 receptor stimulation, through Gαolf signaling, increases cyclic nucleotide (cAMP) formation, ultimately producing transcription factor activation and increasing gene expression [1,6]. Conversely, D2 receptor stimulation decreases these activities through Gαi signaling. The net effect on gene regulation thus reflects an integration of various inputs and intrinsic mechanisms (e.g., PDE activity) [13].

Our findings demonstrate that increasing cAMP signaling via inhibition of the cyclic nucleotide-degrading enzyme PDE10A dramatically upregulates striatal gene expression. These results are consistent with and extend previous findings on the effects of PDE manipulations (inhibition, gene deletion) on such gene expression (e.g., [10,11,27,28]). Moreover, our findings show that there is a close positive relationship between local PDE10A function (basal expression) and gene regulation for the markers of both D1- and D2-MSNs. Thus, PDE10A regulates gene expression in both projection neuron subtypes. This finding is in agreement with the effects of PDE10A inhibition on other cellular functions [7,8] in both neuron types [29]. The affected genes included those encoding the neuropeptides dynorphin (D1-MSNs) and enkephalin (D2-MSNs), as well as immediate-early genes (e.g., zif268), which are all well-established synaptic activity-regulated genes. Similarly impacted was the 5-HT1B serotonin receptor subtype, which is expressed in both projection neuron types [24] and is also dynamically regulated by dopamine and other inputs (cf. [14,18]). This effect was in marked contrast to the 5-HT2C receptor, which was not affected by drug treatments [14,18] and was also not affected by PDE10A inhibition (present findings).

PDE10A is highly enriched in striatal neurons, but is also present at low levels in the cortex and other brain areas [2]. Since it was administered systemically, the PDE10A inhibitor could thus conceivably also have impacted cortical and other inputs to the striatum. Given the tight positive correlation between the PDE10 expression in the striatum and the local changes in gene regulation in the striatum, our results indicate a role for local striatal PDE10A in local gene regulation.

### 4.3. Changes in Gene Expression after Dopamine Depletion: Relationship to Basal PDE10A Expression for D2-, but Not D1-, MSN Markers

A great body of work has determined how dopamine depletion (by 6-OHDA or other neurotoxins) and subsequent L-DOPA treatments alter the gene expression in striatal projection neurons (e.g., [30]). Our findings on the lesion-induced changes in the expression of dynorphin, enkephalin and 5-HT1B are described in detail in our previous papers [14,31] and are in agreement with the earlier literature. We previously also reported on the overall topography (regional variations) of these changes in the striatum, with maximal changes in the sensorimotor sectors, intermediate changes in the associative sectors and minimal changes in the limbic sectors [14,31]. Our present new correlation analysis (scatter plots) further illustrates these regionally differential effects.

Dopamine depletion alone produces an increased expression of enkephalin (and 5-HT1B [24]) in D2-MSNs. Enkephalin expression is especially sensitive to cortical input [32], and the increased expression after dopamine depletion is considered to reflect the effect of disinhibited glutamate inputs (in interaction with other neurotransmitters, for example, acetylcholine [33,34]), due to the loss of inhibitory D2 receptor tone in these neurons (see [13]). Our present findings demonstrate that, after dopamine depletion alone, the increases in the enkephalin and 5-HT1B expression were overall also positively correlated with local levels of PDE10A expression, suggesting a role for PDE10A in these molecular changes in D2-MSNs as well. Decreases in the expression of PDE10A (Ref. [25] and present results) and resulting increases in cAMP signaling [25] after dopamine depletion may thus contribute to such increased gene expression.

In marked contrast to these positive correlations between local PDE10A expression and the enkephalin or 5-HT1B expression, the decreases in dynorphin expression (D1-MSNs) after dopamine depletion, considered to reflect decreased activity in D1-MSNs after the loss of stimulatory D1 receptor activity [21], were unrelated to local PDE10A expression, suggesting no contribution of PDE10A to these effects. 

### 4.4. Changes in Gene Expression Induced by L-DOPA Treatment after Dopamine Depletion: Relationship to Basal PDE10A Levels for D1- and D2-MSNs

Repeated L-DOPA treatment after dopamine depletion produces increased gene expression predominantly in D1-MSNs (e.g., [24,30]), and our findings of increased dynorphin, zif268 and 5-HT1B expression [24] in D1-MSNs are thus also in agreement with the literature (cf. [14]). The minor further increase in enkephalin expression (D2-MSNs) is likely attributable to enhanced re-entrant activity from the cortex (or thalamus) (see [14] for discussion). L-DOPA-induced gene regulation displays distinct regional variations, with maximal effects in the lateral sensorimotor striatum (see [14,31]). Our results show positive correlations between local PDE10A expression and L-DOPA-induced increases in gene expression for dynorphin, enkephalin, zif268 and 5-HT1B. The effects in D1-MSNs (dynorphin, zif268 and 5-HT1B [24]) were more tightly correlated with local PDE10A expression, supporting local regulation. The weaker correlation for enkephalin is consistent with a contribution of indirect (network-level) effects (see above). Nevertheless, the positive correlations between these changes and local PDE10A levels indicate an involvement of PDE10A function in gene regulation for both subtypes of projection neurons, also with L-DOPA treatment.

The mechanisms of striatal gene regulation after dopamine depletion, followed by a repeated L-DOPA treatment, are more complex. For example, after dopamine depletion, the principal driver for dopamine agonist-induced gene regulation is considered to be “supersensitive” D1 receptor signaling [9]. This signaling includes altered receptor internalization (involving GRK6 and β-arrestin [6]), increased G-protein coupling, up-regulated Gαolf function [35], overexpression of adenylyl cyclase, as well as abnormal engagement of other signaling molecules in the D1 receptor-activated second messenger pathways (e.g., [6,9,30,36,37]).

While a considerable amount of information on the function of the above signaling molecules that positively link D1 receptor stimulation to gene regulation has been worked out (see above), their regional distribution in the striatum has not been mapped in any detail, as far as we know. Most molecules seem to have a “fairly uniform” distribution throughout the striatum, with perhaps somewhat higher levels laterally (e.g., [35,38,39]). However, at least some molecules are also regulated by post-transcriptional modifications and phosphorylation [1,37], which would make mRNA mapping unreliable. For example, levels of the Gαolf protein, which is considered the “rate-limiting” step in D1 receptor-activated signaling cascades [37], seem to be regulated by use (degradation) rather than expression [35,39]. In contrast, by demonstrating a close relationship between the PDE10A mRNA levels and dopamine agonist-induced gene regulation, our findings indicate that, for PDE10A, the magnitude of expression is an important determinant for PDE10A function and related gene regulation.

### 4.5. Changes in Psychostimulant-Induced Gene Expression: Relationship to Basal PDE10A Levels

In order to determine the generality for the observed relationship between PDE10A and gene regulation, we next investigated psychostimulant-induced gene regulation in intact adolescent rats. Psychostimulants, including cocaine and methylphenidate, change the expression of hundreds of genes, which occurs also predominantly in the D1-MSNs, and these effects are similar in adolescents and adults [12,13]. We assessed psychostimulant effects on the immediate-early gene zif268 and the neuropeptide substance P, both of which are induced in D1-MSNs [12]. Our results demonstrated a similar positive correlation between the basal PDE10A expression and psychostimulant-induced gene regulation (in adolescents), as described above for gene regulation by L-DOPA after dopamine depletion (in adults). Overall, although statistically significant, these correlations tended to be somewhat less robust than those observed after dopamine depletion plus L-DOPA treatment. This was likely due to a combination of a lower response magnitude in the intact striatum (as opposed to the enhanced responses with the stimulation of supersensitive dopamine receptors after dopamine depletion) and more robust indirect effects (e.g., cortical input [13]) with these dopamine reuptake inhibitors. Nevertheless, this direct relationship between local PDE10A expression and gene regulation induced by these psychostimulants also highlighted the considerable regionally differential gene regulation, as observed before [12,13]: maximal in the sensorimotor striatum, more limited in the associative striatum and modest in the limbic striatum.

## 5. Functional Considerations and Conclusions

Our findings demonstrate a strong positive relationship in rats between the basal expression of PDE10A and gene induction by indirect dopamine agonists, both after dopamine depletion and in the intact striatum. Given that PDE10A functions to counteract (degrade) cyclic nucleotide signaling and thus inhibit cyclic nucleotide-mediated gene regulation, this observed relationship exposes PDE10A as an important component of the second messenger machinery underlying drug/synaptic activity-driven gene regulation in the striatal region. For one, these results indicate that PDE10A serves as a marker to identify striatal regions with a high potential for gene regulation-mediated neuroplasticity in response to synaptic input/drug action. The most affected striatal sectors in the rat—the sensorimotor sectors—correspond to the putamen in primates, suggesting that putamen-based striatal functions may be most at risk (e.g., as seen in Parkinson’s disease). Moreover, PDE10A may serve as a potential target for modifying such drug-induced gene regulation and related neuroplasticity.

## Data Availability

The data generated during the current study are available from the corresponding author on reasonable request.

## References

[1] Girault J.A., Greengard P., Nairn A.C., Steiner H., Tseng K.Y. (2017). Regulation of striatal signaling by protein phosphatases. Handbook of Basal Ganglia Structure and Function.

[2] Kelly M.P., Adamowicz W., Bove S., Hartman A.J., Mariga A., Pathak G., Reinhart V., Romegialli A., Kleiman R.J. (2014). Select 3’,5’-cyclic nucleotide phosphodiesterases exhibit altered expression in the aged rodent brain. Cell Signal.

[3] Schmidt C.J., Chapin D.S., Cianfrogna J., Corman M.L., Hajos M., Harms J.F., Hoffman W.E., Lebel L.A., McCarthy S.A., Nelson F.R. (2008). Preclinical characterization of selective phosphodiesterase 10A inhibitors: A new therapeutic approach to the treatment of schizophrenia. J. Pharmacol. Exp. Ther..

[4] Grauer S.M., Pulito V.L., Navarra R.L., Kelly M.P., Kelley C., Graf R., Langen B., Logue S., Brennan J., Jiang L. (2009). Phosphodiesterase 10A inhibitor activity in preclinical models of the positive, cognitive, and negative symptoms of schizophrenia. J. Pharmacol. Exp. Ther..

[5] Beck G., Maehara S., Chang P.L., Papa S.M. (2018). A selective phosphodiesterase 10A inhibitor reduces L-Dopa-induced dyskinesias in Parkinsonian monkeys. Mov. Disord..

[6] Spigolon G., Fisone G. (2018). Signal transduction in L-DOPA-induced dyskinesia: From receptor sensitization to abnormal gene expression. J. Neural Transm..

[7] Threlfell S., Sammut S., Menniti F.S., Schmidt C.J., West A.R. (2009). Inhibition of phosphodiesterase 10A increases the responsiveness of striatal projection neurons to cortical stimulation. J. Pharmacol. Exp. Ther..

[8] Padovan-Neto F.E., Sammut S., Chakroborty S., Dec A.M., Threlfell S., Campbell P.W., Mudrakola V., Harms J.F., Schmidt C.J., West A.R. (2015). Facilitation of corticostriatal transmission following pharmacological inhibition of striatal phosphodiesterase 10A: Role of nitric oxide-soluble guanylyl cyclase-cGMP signaling pathways. J. Neurosci..

[9] Cenci M.A., Konradi C. (2010). Maladaptive striatal plasticity in L-DOPA-induced dyskinesia. Prog. Brain Res..

[10] Kleiman R.J., Kimmel L.H., Bove S.E., Lanz T.A., Harms J.F., Romegialli A., Miller K.S., Willis A., des Etages S., Kuhn M. (2011). Chronic suppression of phosphodiesterase 10A alters striatal expression of genes responsible for neurotransmitter synthesis, neurotransmission, and signaling pathways implicated in Huntington’s disease. J. Pharmacol. Exp. Ther..

[11] Gentzel R.C., Toolan D., Roberts R., Koser A.J., Kandebo M., Hershey J., Renger J.J., Uslaner J., Smith S.M. (2015). The PDE10A inhibitor MP-10 and haloperidol produce distinct gene expression profiles in the striatum and influence cataleptic behavior in rodents. Neuropharmacology.

[12] Steiner H., Van Waes V. (2013). Addiction-related gene regulation: Risks of exposure to cognitive enhancers vs. other psychostimulants. Prog. Neurobiol..

[13] Steiner H., Steiner H., Tseng K.Y. (2017). Psychostimulant-induced gene regulation in striatal circuits. Handbook of Basal Ganglia Structure and Function.

[14] Padovan-Neto F.E., Patterson S., Voelkner N.M., Altwal F., Beverley J.A., West A.R., Steiner H. (2020). Selective regulation of 5-HT1B serotonin receptor expression in the striatum by dopamine depletion and repeated L-DOPA treatment: Relationship to L-DOPA-induced dyskinesias. Mol. Neurobiol..

[15] Paxinos G., Watson C. (1998). The Rat Brain in Stereotaxic Coordinates.

[16] Altwal F., Padovan-Neto F.E., Ritger A., Steiner H., West A.R. (2021). Role of 5-HT1A receptor in vilazodone-mediated suppression of L-DOPA-induced dyskinesia and increased responsiveness to cortical input in striatal medium spiny neurons in an animal model of Parkinson’s disease. Molecules.

[17] Steiner H., Kitai S.T. (2001). Unilateral striatal dopamine depletion: Time-dependent effects on cortical function and behavioural correlates. Eur. J. Neurosci..

[18] Van Waes V., Ehrlich S., Beverley J.A., Steiner H. (2015). Fluoxetine potentiation of methylphenidate-induced gene regulation in striatal output pathways: Potential role for 5-HT1B receptor. Neuropharmacology.

[19] Willuhn I., Sun W., Steiner H. (2003). Topography of cocaine-induced gene regulation in the rat striatum: Relationship to cortical inputs and role of behavioural context. Eur. J. Neurosci..

[20] Yano M., Steiner H. (2005). Methylphenidate (Ritalin) induces Homer 1a and zif 268 expression in specific corticostriatal circuits. Neuroscience.

[21] Steiner H., Gerfen C.R. (1998). Role of dynorphin and enkephalin in the regulation of striatal output pathways and behavior. Exp. Brain Res..

[22] Yano M., Steiner H. (2005). Topography of methylphenidate (Ritalin)-induced gene regulation in the striatum: Differential effects on c-fos, substance P and opioid peptides. Neuropsychopharmacology.

[23] Seeger T.F., Bartlett B., Coskran T.M., Culp J.S., James L.C., Krull D.L., Lanfear J., Ryan A.M., Schmidt C.J., Strick C.A. (2003). Immunohistochemical localization of PDE10A in the rat brain. Brain Res..

[24] Heiman M., Heilbut A., Francardo V., Kulicke R., Fenster R.J., Kolaczyk E.D., Mesirov J.P., Surmeier D.J., Cenci M.A., Greengard P. (2014). Molecular adaptations of striatal spiny projection neurons during levodopa-induced dyskinesia. Proc. Natl. Acad. Sci. USA.

[25] Giorgi M., Melchiorri G., Nuccetelli V., D’Angelo V., Martorana A., Sorge R., Castelli V., Bernardi G., Sancesario G. (2011). PDE10A and PDE10A-dependent cAMP catabolism are dysregulated oppositely in striatum and nucleus accumbens after lesion of midbrain dopamine neurons in rat: A key step in parkinsonism physiopathology. Neurobiol. Dis..

[26] Gerfen C.R., Surmeier D.J. (2011). Modulation of striatal projection systems by dopamine. Annu. Rev. Neurosci..

[27] Strick C.A., James L.C., Fox C.B., Seeger T.F., Menniti F.S., Schmidt C.J. (2010). Alterations in gene regulation following inhibition of the striatum-enriched phosphodiesterase, PDE10A. Neuropharmacology.

[28] Wilson J.M., Ogden A.M., Loomis S., Gilmour G., Baucum II A.J., Belecky-Adams T.L., Merchant K.M. (2015). Phosphodiesterase 10A inhibitor, MP-10 (PF-2545920), produces greater induction of c-Fos in dopamine D2 neurons than in D1 neurons in the neostriatum. Neuropharmacology.

[29] Nishi A., Kuroiwa M., Miller D.B., O’Callaghan J.P., Bateup H.S., Shuto T., Sotogaku N., Fukuda T., Heintz N., Greengard P. (2008). Distinct roles of PDE4 and PDE10A in the regulation of cAMP/PKA signaling in the striatum. J. Neurosci..

[30] Cenci M.A., Steiner H., Tseng K.Y. (2017). Molecular mechanisms of L-DOPA-induced dyskinesia. Handbook of Basal Ganglia Structure and Function.

[31] Altwal F., Moon C., West A.R., Steiner H. (2020). The multimodal serotonergic agent vilazodone inhibits L-DOPA-induced gene regulation in striatal projection neurons and associated dyskinesia in an animal model of Parkinson’s disease. Cells.

[32] Uhl G.R., Navia B., Douglas J. (1988). Differential expression of preproenkephalin and preprodynorphin mRNAs in striatal neurons: High levels of preproenkephalin expression depend on cerebral cortical afferents. J. Neurosci..

[33] Pisani A., Bernardi G., Ding J., Surmeier D.J. (2007). Re-emergence of striatal cholinergic interneurons in movement disorders. Trends Neurosci..

[34] Reynolds J.N.J., Avvisati R., Dodson P.D., Fisher S.D., Oswald M.J., Wickens J.R., Zhang Y.F. (2022). Coincidence of cholinergic pauses, dopaminergic activation and depolarisation of spiny projection neurons drives synaptic plasticity in the striatum. Nat. Commun..

[35] Hervé D., Lévi-Strauss M., Marey-Semper I., Verney C., Tassin J.P., Glowinski J., Girault J.A. (1993). G(olf) and Gs in rat basal ganglia: Possible involvement of G(olf) in the coupling of dopamine D1 receptor with adenylyl cyclase. J. Neurosci..

[36] Gerfen C.R., Steiner H., Tseng K.Y. (2010). D1 dopamine receptor supersensitivity in the dopamine-depleted striatum: Aberrant ERK1/2 signaling. Handbook of Basal Ganglia Structure and Function.

[37] Hervé D. (2011). Identification of a specific assembly of the g protein golf as a critical and regulated module of dopamine and adenosine-activated cAMP pathways in the striatum. Front. Neuroanat..

[38] Sakagami H., Sawamura Y., Kondo H. (1995). Synchronous patchy pattern of gene expression for adenylyl cyclase and phosphodiesterase but discrete expression for G-protein in developing rat striatum. Mol. Brain Res..

[39] Hervé D., Le Moine C., Corvol J.C., Belluscio L., Ledent C., Fienberg A.A., Jaber M., Studler J.M., Girault J.A. (2001). Galpha(olf) levels are regulated by receptor usage and control dopamine and adenosine action in the striatum. J. Neurosci..

